# Dual-Processing Altruism

**DOI:** 10.3389/fpsyg.2013.00193

**Published:** 2013-04-18

**Authors:** Suna Pirita Kinnunen, Sabine Windmann

**Affiliations:** ^1^Allgemeine Psychologie II, Department of Psychology, Johann Wolfgang Goethe UniversityFrankfurt am Main, Germany

**Keywords:** dual-processing, intuition, altruistic punishment, volunteering, donation, moral courage

## Abstract

Altruism refers to an other-benefiting behavior that is costly but bears no direct profit to oneself. At least three different forms can be distinguished: help giving, altruistic punishment, and moral courage. We investigated the differential impact of two thinking modes, intuitive (System 1) and rational (System 2), on these three altruistic behaviors. Situational (state-related) thinking style was manipulated via experimental instructions and generally preferred thinking style (trait-related) was assessed via questionnaires. We found that of the subjectively preferred thinking styles (trait), faith in intuition (System 1) promoted sharing and altruistic punishment, whereas need for cognition (System 2) promoted volunteering in a situation that required moral courage. By contrast, we did not find a significant effect of situational thinking style (state) on any of the altruistic behaviors, although manipulation checks were positive. Results elucidate the affective-motivational underpinnings of different types of altruistic behaviors.

## Introduction

Altruism is a form of prosocial motivation that benefits others but is costly to oneself. This can mean that the benefit to others is greater than the benefits to oneself, or in strictest terms that there are no benefits to oneself. In terms of overt behavior, altruism can take at least three forms: costly sharing, altruistic punishment, and moral courage. These three have been investigated rather independently in the past; hence it is not clear whether they originate from the same motivation. The first form of altruism is *costly sharing* and it refers to help giving, when one party gives from their resources to another without receiving anything in return. Abundant research has shown that the most often mentioned motivator this form of altruism is empathy (e.g., Batson, 1991), and it is thought to be supported by and serves to maintain positive affect (Stel et al., 2008).

Secondly, human altruism can take the form of costly punitive actions against norm violators with the aim of enforcing social norms, which is referred to as *altruistic punishment*. This behavior has been suggested to be crucial to encouraging and maintaining social cooperation (Fehr and Gächter, 2002), and to increase equality among group members (Fowler et al., 2005). The emotive-motivational basis includes feelings of anger aroused by the observed unfairness (Pillutla and Murnighan, 1996; Fehr and Gächter, 2002; Singer and Steinbeis, 2009), and feelings of satisfaction when norm violators are punished (de Quervain et al., 2004; Strobel et al., 2011). While clearly altruistic punishment is costly to oneself, the benefit to others may not be as straight forward; altruistic punishment maintains fairness and cooperation among humans, and can thus be seen as a social investment benefiting societal groups at large, especially under anonymous conditions. In anonymous situations, the benefit to oneself in form of reputation building is eliminated as is the possibility of retaliation or social sanctions.

Finally, there is *moral courage*, which is the willingness to speak up or take action in a situation that conflicts with one’s moral or feelings of justice. Justice sensibility, moral mandates, and anger seem to be among the promoters of moral courage (Kayser et al., 2010). Previous studies have suggested that anticipated emotions and self-conscious emotions – such as guilt, shame, and pride – mediate the willingness to engage in this type of potentially costly act against injustice (Sekerka and Bagozzi, 2007; Sekerka et al., 2009). In moral courage situations, as in altruistic punishment, the benefit to others can be more indirect than in help giving and sharing behavior as the benefiting party may not be a particular person or group. Instead, the benefit can go to groups of people or to society at large in the form of establishment of defense of higher moral values. Many times, however, there will also be a directly benefiting party such as an individual or a minority of people whose rights have been violated, for example in a bullying situation.

From the description of the three forms of altruism it seems apparent that when people are in certain affective states, their altruistic behaviors should be stronger than when they are in a neutral state. This could mean that highly emotional and emotion-driven individuals, those relying more on their intuition, could be more prone to act altruistically than people less reliant on their emotions. Specifically, more empathetic individuals should show stronger help giving and sharing behavior, and those who tend to develop feelings of anger should be more inclined to punish and offensively confront violators of social norms or moral standards as an instantiation of altruistic punishment and moral courage.

However, the case might be different under non-reciprocal and anonymous conditions. When no direct social interaction is taking place, so that the consequences of the altruistic acts are not directly observable, cognitive control processes may be required to pursue other-benefiting behavior in accordance with one’s own moral and fairness standards. This would include the need to inhibit affective or egoistic impulses in favor of uncertain, delayed, and impersonal benefits (e.g., for society as a whole). Under those circumstances it might be that people who like to think things through and consider distant and long-term consequences of their actions before making a decision might engage more in prosocial behaviors, despite lower empathy.

The distinction between intuitive and rational modes of processing is at the core of dual-processing theories which differentiate, in the most neutral terms, between System 1 and System 2 (Kahneman and Frederick, 2002; Evans, 2003). Most of the authors agree that System 1 processes are unconscious, automatic, and rapid and those of System 2 are conscious, slow, and deliberative (for a review, see Evans, 2008). Many different types of manipulations have been devised to study these processes (e.g., Stanovich and West, 1998; Cohen and Andrade, 2004; Horstmann et al., 2009). For example, it has been established that knowledge about the rules of a task and asking people to deliberate allows them to override an intuitive judgment in that task (Kahneman and Tversky, 1982). However, whether people apply the information to override the intuitive processing depends, among others, on their cognitive skills and on the formulation of the task that make the rules apparent (Kahneman and Frederick, 2002). As of yet there are no empirical investigations into the question of whether and how these processing modes affect all of the three types of altruistic behavior described above, and in what way. A recent study has focused on a connection between situational processing mode and social cooperation (Rand et al., 2012) by investigating the connection between reaction times and cooperation in economic games. Although the study had asked “whether people are predisposed toward selfishness,” predisposition in the sense of individual aptness was actually not differentiated from situational factors. We set out to make that differentiation.

The aim of this study is to investigate to which extent emotional-intuitive (System 1) relative to rational cognitive processing (System 2) contributes in anonymous situations to the three different types of altruistic behavior: help giving, altruistic punishment, and moral courage. This goal is pursued at two levels within the same study: first, the influence of actual situational processing mode is investigated by experimental manipulation of thinking style via instructions; we refer to this as the *state processing mode*. Second, the influence of subjectively preferred thinking style is investigated by means of individual assessment via questionnaire; we refer to this as the *trait processing mode*. The state-related processing mode is thought to be more situational and contextually activated, and hence more related to flexible top-down control than the latter. The trait-related processing should be habitual and therefore quite automatic in nature. It could be thought as the preferred processing style over time that the situational factors influence and variate. Although at the forefront of current discussions (Kahneman, 2011), the relationship between the rational versus intuitive thinking style at those two levels of processing (state versus trait) and their impact on prosocial behavior has not to our knowledge been examined before. Results will provide us with important insights into the mechanisms through which altruistic behavior is accomplished and can be enhanced.

To approach the important question of altruism under conditions where reciprocity is not possible, we attempted to create a setting where altruistic acts are performed toward anonymous others. This makes the research question more challenging as it bears upon the “true” altruism motice (Harbaugh et al., 2007). In addition, the anonymity of the situation should degrease any interpersonal effects, such as the effect of gender, age, likeability, or other attributes of the other in the monetary games or reputation building, retaliation, or social sanctions or rewards in any of the measures. In addition, we had participants make decisions about considerable amounts of money to increase the impact of their acts and to make the decisions feel as real as possible. To warrant external validity, we assessed all three forms of altruism strictly with behavioral tasks. Costly sharing was assessed via dictator game (DG), where the participant was asked to split an amount of money between her and an anonymous other (see, e.g., Bolton et al., 1998), and via donating to charity. Altruistic punishment was assessed via a series of ultimatum games (UG), where the participant decided whether to accept a split an anonymous other had made, resulting in a varying amount of money for them both, or to reject the offer in which case neither (the participant nor the anonymous other) would get anything (see, e.g., Thaler, 1988; Nowak et al., 2000). Next the participant was asked to volunteer in a task that requires moral courage. When leaving the laboratory, the participants were pointed to a charity poster advert and offered an opportunity to donate. The donation was meant to assess costly sharing. Previous studies from the embodied cognition approach and attribution research have shown that keeping such manipulations incidental is vital to their behavioral effects (Schnall et al., 2008, 2010).

We hypothesize that the pattern of rejecting unfair offers in the UG will be stronger for participants in which intuitive decision-making (System 1) is encouraged compared to participants in which rational cognitive decision-making (System 2) is encouraged. Likewise, in the moral courage measure, we anticipate that participants in the intuition condition will behave more altruistically than in the rational condition. In the two other decisions (DG and donation), both of which address help giving, we predict that intuitive processing leads to higher levels of altruism than the rational mode. The effects of both state and trait processing modes are hypothesized to be in the same direction.

## Materials and Methods

### Participants

Forty-eight (*N* = 29 female) participants, mean age 23.25 years (SD = 5.85 years), were recruited from lectures of the University and from the audience of the Night of Science in Frankfurt. Participants were students of Medicine (*N* = 18), Economics (*N* = 9), Natural Sciences and/or Mathematics (*N* = 9), or other disciplines (*N* = 12). No students of Psychology were included. The study was approved by the ethical committee of psychology Johann Wolfgang Goethe University, Frankfurt am Main. All the participants gave their written informed consent before the experiment.

### Materials

The short version of the Rational-Experiential Inventory (REI-10; Pacini and Epstein, 1999) was used as the measure of generally preferred thinking style. The REI-10 includes two independent subscales: faith in Intuition (FI; e.g., “My initial impressions of people are almost always right.”) and Need for Cognition (NFC, e.g., “I would prefer complex to simple problems.”). The inventory gives a distribution of the participants’ preferred thinking styles and the items were rated on a five-point rating scale (1 = *completely false* to 5 = *completely true*). The inventory was translated by a native German speaker and checked by another. The Cronbach’s alpha reliability was 0.60 for both of the five-item measures of FI and Need for Cognition. The moderate degree of consistency was likely due, in part, to the limited number of participants.

The Measure of Emotional Empathy (Mehrabian and Epstein, 1972) was used to acquire a base-line of the participants’ tendency to react with empathy. The questionnaire consists of 33 items (e.g., “Seeing people cry upsets me.”) rated on a nine-point scale (4 = *very strong agreement* to −4 = *very strong disagreement*). The questionnaire was translated by a native German speaker and checked by another. The Cronbach’s alpha reliability of the 33-item measure of emotional empathy was 0.70.

The Barratt Impulsiveness Scale (BIS-15; Spinella, 2007) was used to assess impulsiveness. The 15-items of the scale were assessed on a four-point scale ranging from *Seldom or never* to *Nearly always or always*. A German version of the scale was used (Meule et al., 2011). The Cronbach’s alpha reliability of the 15-item measure of impulsiveness was 0.78.

To measure everyday altruism, a modified altruism questionnaire by Rushton et al. (1981) was used. The scale was translated by a native German speaker and checked by another. Eight items from Rushton et al.’s scales were chosen with two new items [“I have bought someone an ‘immaterial gift’ or donated money to a charity (e.g., Greenpeace, WWF, Unicef) in their name” and “I have ignored a person who has asked me the way or to make change for their money”]. Three items were rated on a seven-point scale ranging from *Never* to *Daily* and the rest on a five-point scale ranging from *Never* to *Every time*. The internal consistency of the scale was not very high, Cronbach’s α = 0.56.

Social Value Orientation (SVO; Murphy et al., 2011) Slider Measure assesses how much concern a person has for others. It consists of six primary and nine secondary items where the participant indicates by marking on a continuum their resource allocation choice. The measure gives an angle, which indicates the participant’s social value orientation, and it can be categorized as follows: greater than 57.15° altruistic, between 22.45° and 57.15° prosocial, between −12.04° and 22.45° individualist, and less than −12.04°competitive. A German version of the measure was used. The answers of all except for one participant were transitive.

Inclusion of Other in Self (IOS) Scale (Aron et al., 1992) consists of Venn-like diagrams representing different degrees of overlap of two circles and measures the degree of interpersonal closeness. In addition to the seven used by Aron et al. (1992), we included a diagram where the two circles are clearly separate. The participants were asked to select the diagram that best described their relationship to (a) other people in general, (b) people they know, but are not friends with, (c) their closest friend, (d) their spouse, and (e) their closest blood relative. In contrast to the circles Aron et al. (1992) introduced, in our study the circles did not vary in size across response alternatives. This was done to keep the notion of the “size of the self” constant. Also, in the preliminary testing of the questionnaire, people noted the change of the circle sizes and wondered about its meaning. The scale was constructed by a native German speaker and checked by another. The items were used separately in the analysis.

A set of logical dilemmas were used as a manipulation check. The logical dilemmas consisted of the Cognitive Reflection Test (CRT; Frederick, 2005), a new question (“How much does a brick weigh if it weighs 1 kg and a half a brick?”) and a set of four syllogisms (e.g., Zielinski et al., 2010). In the four categorical syllogisms, the participant was to choose whether the deduction was true or false; the first and the last of them were true. The second and the fourth were adaptations from a study by Zielinski et al. (2010) who reported the percentage of correct answers as 0.20 and 0.53 respectively. The measures were constructed or translated by a native German speaker and checked by another. The syllogisms were as follows:

- All square blocks are green blocks. Some big blocks are square blocks. ⇒ Some big blocks are green blocks.
- Some tables are not wooden tables. No wooden table is yellow. ⇒ Some tables are not yellow.
- All square blocks are green blocks. Some big blocks are not square blocks. ⇒ Some big blocks are not green blocks.
- Some round tables are wooden. No wooden table is big. ⇒ Some round tables are not big.

Delay of gratification was measured with four questions, also used by Frederick (2005). The questions were as follows:

- Would you prefer to get 3400 Euros this month or 3800 Euros next month?
- Would you prefer to get 100 Euros now or 140 Euros next year?
- How much would you be willing to pay for an overnight shipping of a chosen book?

Participants were presented three dilemmas where they had to decide how morally acceptable it would be to kill one person to save a number of other people. In the first dilemma, the participant was asked to rate on a six-point Likert-scale how permissible it is for a submarine captain to shoot a bleeding crew member in order to save oxygen, and thus save the rest of the crew (for the full text, see Paxton et al., 2012). The two remaining dilemmas are two versions of the Railroad dilemma. In the first version, the participant rates how permissible it is to flip a switch to redirect a train to another track killing only one man instead of five and in the second, to push a pedestrian to the railroad tracks and make the train stop again killing only one man instead of five (see, e.g., Greene et al., 2001). The dilemmas were translated by a native German speaker and checked by another. The dilemmas were analyzed separately. Previous research has found that the manipulation of one’s thinking mode affects the judgments they make on moral dilemmas (Paxton et al., 2012).

### Procedure

The experiment followed a 2-factorial design (between groups). Upon entering the experiment room the participants were randomly assigned into one of two groups: System 1 group was instructed to decide according to their first impulse, their gut-feeling and intuition and System 2 group was instructed to deliberate and take their time before deciding. The maximum pay-off the participants could obtain was 80 Euros (paid in cash). Two female assistants who were not aware of the hypotheses collected the data. The duration of the entire experiment was less than 1 h.

At the beginning of the experiment, before manipulations, the participants were asked to fill out electronic versions of the REI-10, a questionnaire of everyday altruism based on the altruism questionnaire by Rushton et al. (1981), and the Measure of Emotional Empathy and paper versions of the SVO and the IOS.

After filling out the questionnaires, half of the participants were assigned into System 1, intuitive condition, and the other half into System 2, rational condition. The System 1 condition obtained the following instruction: “Relax and make your decisions as quickly as possible. Trust your intuition and answer by your gut-feeling. Give the first choice that comes to your mind.” The System 2 condition got the following instruction: “Take your time and think your choices through before you make your decision. Do not to let emotions get in the way. Deliberate on your answer before giving it.” The instructions were repeated before each of the following phases of the experiment.

Participants were given 20 Euros in cash (coins and notes) and asked to split the money with an unknown participant in a following experiment (DG). They were told that they can give anything between all of the money to nothing at all and that the choice is completely theirs to make. The variable DG derived from the DG was the amount of money the participant kept to herself. After this one-shot DG, the participant was given 2 Euros in cash and was again asked to split the money. This time she was explained that the other person has the opportunity reject the offer, in which case neither of them will receive anything. The participant was told that using a list of minimum offers that previous participants found to be just acceptable, the experimenter would randomly assign her offer to one of those participants, and accept or reject it accordingly. This was meant to increase the credibility of the game and to suggest that the decision the participant makes will have real consequences. In reality, all offers that were greater than zero were accepted (no offers of zero were made). Before making their decision, the participant was asked to repeat the instruction to the experimenter in order to ensure full understanding of the game. The UG was then repeated with 18 Euros. The variables derived from these UG were the amounts of money the participants received from the 2 Euro split UG1 and from the 18 Euro split UG2.

**Figure 1:**
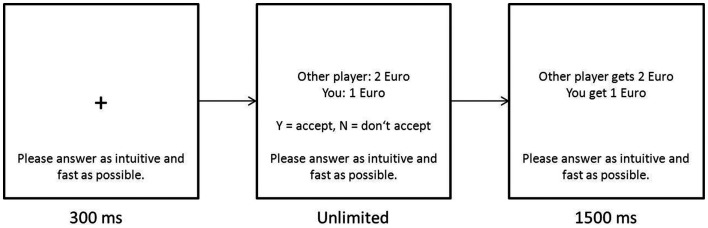
**The design of the computerized ultimatum game**.

After the two trials of the UG in the role of the proposer, the participant played 20 trials in the role of the responder with different proposers on a computer. The participant was presented with a short explanation of an UG with an example. She was told that the offers had been made by previous participants and that her decisions will have true consequences in following experiments. In reality, the offers were predetermined, and the same for each participant, though presented in randomized order. She was also told that her responses in the UG would be saved in a database and used later on as the responses to future participants’ proposals. The participant was also told that the pay she would receive is the sum of all accepted offers. The design of the game is presented in Figure 1 and the Euro amounts of the offers in Table 1. The variable derived from the computerized UG was the sum their winnings UG3.

**Table 1:** **Euro amounts offered by proposers in the computer ultimatum game**.

11%	15%	20%	40%
0.13	0.76	0.40	1.30
2.16	7.33	13.00	1.00
0.50	1.00	0.10	0.25
3.50	0.50	1.50	2.50
0.25	1.50	2.00	0.30

*The proposer’s splits are 100 − *x*%. For example, the first split is 0.13 Euro for the participant and 1.05 Euros for the proposer*.

After 20 trials of single-shot UGs, the participant was given the money she earned and was asked to fill out a series of questionnaires. First, she was asked to rate on a eight-point scale from “Very unfair” to “Very fair” the fairness of five different offers in the UG, namely 0.13 (11%), 1 (15%), 1.3 (40%), 2.16 (11%), and 0.1 Euros (20%). This was followed by the logical dilemmas. Lastly, the participant was to rate the acceptability of actions in the three moral dilemmas.

After the dilemmas, the participant was thanked and made believe the experiment had ended. However, just before leaving the lab, she was told that we had agreed to some colleagues from another faculty to spread information of a campaign for which they are searching for participants. She was given a flyer and told that this was about volunteering as a discussion group leader in a project aimed at teaching tolerance toward foreigners to young delinquents found guilty of racist crimes. The information given was meant to make volunteering intimidating; facing the young criminals in such a situation and trying to make them more tolerant would require moral courage (Kayser et al., 2010). Directly after the participant had made her decision, she was thoroughly debriefed and asked to rate (five-point Likert-scale) how threatening and uncomfortable leading the discussion group would have been.

After the debriefing, on the way out of the laboratory, the participant was incidentally made aware of an actual collection currently running in the department and she was pointed to a charity poster advert from Greenpeace and a charity collection box, where she could make a voluntary donation if she liked to contribute. Greenpeace’s work against oil industry was selected for its visibility in the media and provoking advertisement showing a burning oil platform. This measure represents an incidental and thus ecologically valid measure insofar as it is hopefully not recognized as part of the experimental procedure by the participant. After the participant left, the collection box was checked and the amount of the donation taken as a measure. The participant was then sent a debriefing e-mail disclosing the purpose of the donation, ensuring that all of the money donated would be given to Greenpeace, and including an overview of the goals of the experiment.

## Results

The distributions of most of the dependent variables differed significantly from normal, and hence mainly non-parametric methods were used: Spearman’s rank correlation coefficient for correlation and Mann–Whitney–Wilcoxon (MWW) for group comparisons. From the independent measures, the Need for Cognition, the Empathy scale, and the SVO agreed with a normal distribution, NFC: Shapiro–Wilk *W*(38) = 0.97, *p* = 0.348; Empathy: *W*(38) = 0.97, *p* = 0.382; SVO: *W*(38) = 0.96, *p* = 0.198. For these three scales, parametric analyses (Pearson’s product-moment correlation and Student’s *t*-test for group comparisons) were used when appropriate. As the estimates of effect size, we use Cohen’s *d* (Cohen, 1969) for the parametric and *r* for the non-parametric comparisons (Cohen, 1969; Fritz et al., 2012) in order to present the most commonly known and studied indicators. Even though these measures are not directly comparable, they are, to our knowledge, the most widely applied, and thus offer the best comparability, in their respective areas of application.

### Manipulation check

To check the effectiveness of the manipulation, the sum of the response times in the UG on the computer were calculated and compared between the conditions. The mean time in the System 1 condition was 615.92 s (SD = 208.84 s, Mdn = 577.71 s) and in the System 2 condition 2700.45 s (SD = 4220.45, Mdn = 1539.02 s). The difference between the conditions was significant, MWW *U* = 39.00, *p* < 0.001, and the effect size was large, *r* = 0.74, following Cohen’s (1969) operational definitions of effect sizes.

The second manipulation check was the CRT and the brick question. Due to the similar nature of the two tasks, a composite variable *CRTB* was formed. The Cronbach’s alpha reliability of the CRTB was 0.69. The System 2 group scored higher on CRTB, *M* = 2.33, SD = 0.30, Mdn = 2.50, than the System 1 group, *M* = 1.21, SD = 0.23, Mdn = 1.00. The difference was statistically significant and the effect size was medium, MWW *U* = 162.00, *p* = 0.008, *r* = 0.38.

The two conditions did not differ in the number of correct answers in the four syllogisms, System 1 and System 2: Mdn = 2.00; MWW *U* = 242.00, *p* = 0.276, presumably due to floor effects. The percentage of better than chance (more than half correct) answers was only 33: 10 participants got more than half correct in the System 1 condition and six participants in the System 2 condition.

### State processing mode

To study the effect of situational thinking mode on different types of prosocial behavior, the differences on these measures between the two manipulation conditions were investigated. The mean amount of money kept in the DG was 13.65 Euros (SD = 3.53 Euros; Mdn = 14.00 Euros), in the first UG (UG1) 1.08 Euros (SD = 0.18 Euro, Mdn = 1.00 Euros), and in the second UG (UG2) 10.44 Euros (SD = 3.53 Euros, Mdn = 10.00 Euros). These results agree with previous research (Güth et al., 1982; Thaler, 1988; Strobel et al., 2011). The mean total won in the computerized UG (UG3) was 56.63 Euros (SD = 11.36 Euros, Mdn = 34.15 Euros). No significant differences were found between the two conditions in the decisions on the DG or the UG, DG: MWW *U* = 214.50, *p* = 0.119; UG1: *U* = 222.50, *p* = 0.112; UG2: *U* = 224.00, *p* = 0.175; UG3: *U* = 279.00, *p* = 0.852. The medians of these four games respectively were 12.50, 1.00, 10.00, and 33.37 Euros for the System 1 group and 15.00, 1.00, 11.00, and 34.27 Euros for the System 2 group. Also, the difference of total amount of winnings between the two conditions was not significant, *U* = 241.50, *p* = 0.338.

The difference between the System 1 and System 2 conditions in volunteering as a group leader was not significant, MWW *U* = 264.00, *p* = 0.562. Approximately 46% of the participants in System 1 condition and 38% of participants in System 2 condition volunteered in the moral courage task. The overall percentage of volunteering was 42%. Those who did not volunteer rated the task significantly more unpleasant than those who volunteered, *t*(46) = 3.25, *p* = 0.002, there was no difference in how threatening they rated the task. The effect size of the rating of unpleasantness was large, Cohen’s *d* = 0.97.

The mean amount of money donated by the System 1 group was 0.89 Euro (SD = 2.23 Euro) and by the System 2 group 1.13 Euro (SD = 1.51 Euro). The difference between the groups was not significant, MWW *U* = 280.00, *p* = 0.856. The total amount of donations was 48.42 Euro, which was approximately mere 1.78% of the total amount of winnings. There was no significant difference in the donation rate between the manipulation groups; nearly 45% of the participants made a donation. The largest donation was 9.83 Euros.

### Trait processing mode

To investigate the influence of the generally preferred thinking style on the measures of altruistic punishment and helping behavior, the two subscales of the REI were correlated with the winnings in the DG and UG monetary games and the amount of donation. In addition, correlations of the NFC and the FI with other questionnaire measures and interrelations of the dependent variables were established in order to explore the possible mechanisms by which the generally preferred thinking style could influence social cooperation. The results are presented in Table 2. Importantly, the NFC and the FI did not correlate significantly with each other, Spearman’s *r* = 0.15, *p* = 0.297, consistent with the theoretical construction of the scales as two independent dimensions (Pacini and Epstein, 1999). Results show that the higher the FI, the less money participants kept for themselves in the DG, suggesting higher tendency for sharing behavior, and the less money they accepted as respondents in the computerized UG as the responder, suggesting higher tendency for altruistic punishment. When all the winnings were combined, the higher FI the participant had, the lower his total amount of winnings was.

**Table 2:** **Non-parametric correlations of need for cognition and FI with the dependent variables of social cooperation and with the other questionnaire measures, and their intercorrelations**.

Measure	NFC	FI	2	3	4	5	6	7	8	9
1 DG	−0.08	−0.42**	0.30*	0.64***	0.27^†^	0.01	−0.19	−0.34*	−0.13	−0.71***
2 UG3	−0.10	−0.35*		0.88***	0.50***	−0.08	−0.09	0.15	0.17	−0.18
3 Total of games	−0.16	−0.46**			0.47**	−0.02	−0.19	−0.11	0.03	−0.45**
4 MC	0.31*	−0.23				−0.15	−0.10	0.04	0.26^†^	−0.01
5 Impulsiveness	−0.28^†^	0.37*					0.10	−0.01	−0.03	0.03
6 Empathy	0.06	0.45**						0.34*	0.51**	0.22
7 IOS people known	0.07	0.33*							0.45**	0.28^†^
8 IOS relative	0.19	0.34*								0.32*
9 SVO	0.22	0.34*								

***p < 0.001, **p < 0.01, *p < 0.05, ^†^*p* < 0.10. Correlations of the NFC (need for cognition) with the empathy and the SVO (social value orientation) are parametric. FI, Faith in Intuition, DG, Dictator Game, UG3, computerized Ultimatum Game, MC, Moral Courage task, IOS, Inclusion of Other in Self.

Need for cognition had no significant correlation with the money kept in the DG, the amount of winnings in the computerized ultimatum game, or the combined winnings in the monetary games.

Generally preferred thinking style had an influence on the decision to volunteer as a discussion group leader: those who volunteered in the moral courage task had a higher need for cognition than those who did not volunteer, Volunteers: *M* = 18.05, SD = 2.87; Non-volunteers: *M* = 16.00, SD = 3.45, the difference was statistically significant, *t*(45) = 2.17, *p* = 0.035, and the effect size was medium, *d* = 0.65. A similar trend was found with FI, so that people who volunteered in the moral courage task scored lower in the FI compared to those who did not volunteer, MWW *U* = 205.50, *p* = 0.116, the effect size was small, *r* = 0.22.

Information about the mechanisms behind the relationship between FI and altruistic behaviors can be taken from the intercorrelations with other questionnaires. The higher the FI, the higher the person scored on impulsiveness, empathy, social value orientation, inclusion of others to self in the context of people they know and inclusion of blood relatives to self. Social value orientation correlated significantly negatively with the money kept in the DG, so that the more altruistic participants were in their orientation, the more money they gave in the DG to the anonymous other. The negative relationship between the SVO and the amount of money kept in the DG was significantly stronger than that of FI and money kept in the DG, *z* = 2.08, *p* = 0.019. The effect size of the difference between the correlations was computed as suggested by Siegel and Castellan (1988). The effect size was medium, *q* = 0.44. Interestingly, the SVO did not correlate with the other monetary games, moral courage, or donation to charity.

Need for cognition did not have many significant connections with the prosocial personality measures. There was a trend that the higher a person scored on the impulsiveness scale, the lower they rated their need for cognition, Spearman’s *r* = −0.28, *p* = 0.064. The difference between the correlations of the NFC and the FI with impulsiveness was statistically significant, *z* = −3.21, *p* < 0.001, and the effect size was large, *q* = 0.68. Neither the NFC nor the FI correlated significantly with the amount of money the participant kept in the UG as the proposer or the amount of money donated to charity. No other significant correlations for the NFC or the FI were found.

### Additional results

No significant correlations were found between the sum variable of self-reported everyday altruism and the behavioral measures of altruism (all *p* > 0.44). In order to investigate this further, a correlation between the item “I donate to charity” and the amount of donation made in the experiment was formed. There was a trend that the higher participants rated this item, the more they gave to charity in the experiment, Spearman’s *r* = 0.26, *p* = 0.075. However, the correlation between the everyday altruism score and the SVO was nearly non-existent.

## Discussion

We have studied the relationship between rational cognitive and emotional-intuitive thinking modes in the context of prosocial behavior. We have focused separately on state and trait processing styles and on three different types of altruistic behaviors, namely sharing and helping behavior, altruistic punishment, and moral courage. The main result is that the generally preferred thinking style of FI was linked with altruistic punishment and some form of sharing behavior while that of need for cognition was associated with moral courage.

The situational or state thinking style was manipulated by experimental instructions to either decide by intuition or to deliberate and take time before deciding, an effective method derived from literature (e.g., Kahneman and Tversky, 1982; Horstmann et al., 2009). The manipulation did seem to work, as evidenced by both the manipulation check task and by response times in the computerized UG. Nonetheless, no significant differences between the two groups were found in sharing/help giving, altruistic punishment, moral courage. The effects on the economic games were too small to become significant, and thus failed to replicate the results of a study by Rand et al. (2012) who found that those who have been prompted to answer quickly and intuitively behave more cooperatively in economic games than those who have been instructed to deliberate on their decisions. One reason could be the relatively small sample size which may have kept the significance of the differences too low. Another reason could be that in this study, the amount of money the participants could receive was uncommonly high. The maximum amount of 80 Euros is a substantial sum, especially for students, and it was designed to make the decisions feel real and have true consequences, real impact. However, this may have primed the participants to focus on the money and to leave other facets of the experiment for lesser attention.

However, in addition to manipulating state processing, we investigated the impact of thinking style trait. Here we found a number of significant relationships with FI. Firstly, FI was inversely connected with the amount of money participants received from the monetary games. More accurately, the higher their level of FI, the more money they gave away in the DG (higher sharing behavior), and the less they accepted unfair offers in the computerized UG (altruistic punishment). As individuals with higher FI tend to rely more on their affective states, they appear to decide more prosocially in monetary games compared to individuals low in FI. This is in line with the results of Rand et al. (2012) that suggest a special role of intuition in promoting cooperation, although they focused on the situational processing mode. Part of this connection may be mediated by stronger social bonding and higher emotional care for the wellbeing of other individuals. At the same time, these individuals describe themselves as more impulsive, which might render them more likely to react with feelings of anger when confronted with an unfair offer in the UG, hence they show more altruistic punishment (c.f. Fehr and Gächter, 2002; Singer and Steinbeis, 2009).

By contrast, those low on FI may not respond so emotionally *per se*, or they tend to override their emotive impulses when making monetary decisions. As a result, they punish less when given an unfair offer and share less in the DG, thereby making more money. It thus seems that sharing behavior and altruistic punishment could be two forms of altruism that depend on trait-related intuitive processing.

Contrary to FI, need for cognition was positively associated with volunteering as a discussion group leader in a situation that required moral courage. The higher individuals scored in need for cognition, the more likely they were to find the courage to confront that potentially dangerous situation. Together with the above results, this could represent an empirical dissociation between type of prosocial behavior and trait-related thinking mode. FI seems relevant for helping/sharing behavior and altruistic punishment, but not so much for moral courage, while the opposite may be true for need for cognition. The question is: what motivational basis drives this dissociation?

The crucial difference may be that situations affording moral courage enact feelings of anxiety, and with it a behavioral motivation to withdraw, alongside prosocial feelings (compassion) and anger, two approach-related emotions. Therefore, a behavioral conflict can result especially in prosocial individuals who tend to react more emotionally: on the one hand, they feel like withdrawing from the intimidating situation (e.g., Sekerka and Bagozzi, 2007), on the other hand, their anger fuels them into taking action (e.g., anger, Kayser et al., 2010). The net result of this fight-or-flight conflict may result in very little, while individuals with relatively high need for cognition may either experience much less of this conflict or find sufficient self-discipline or self-regulation in order to override such impulses, and act in line with their reasoning and moral standards.

This view would refine previous suggestions by Kayser et al. (2010) who argue that the specific emotion of anger leads to moral courage. Indeed, anger directed toward the offender should support the moral position and encourage to stand up for one’s beliefs. However, we argue, this process may manifest in behavior only when the opposing anxiety-driven motivation to withdraw (and to avoid conflict with other people) is relatively low or can be controlled, hence we observed it only in individuals who score high in need for cognition. On the other hand, we need to bear in mind that the present experiment contained many different measures of prosocial behavior and it could be that the order of the tasks has influenced the willingness to volunteer as a group leader; participants scoring high in FI may have felt they have already given enough in the previous tasks while participants scoring high in need for cognition may have taken this task as their opportunity to contribute after gaining in the previous tasks. We therefore caution against over interpretation of this particular finding without prior replication.

Help giving in the form of donating to a charity was not connected to any of the generally preferred thinking styles. A low percentage of the participants donated money to charity, and the amounts donated were on average less than 2% of the amount they had won from the monetary games. Notably, this measure was different from the other decisions as the donation was made alone outside the experiment room without any sense of being observed. The participants were left alone in an anteroom of the laboratory, and the money they donated was counted and they were debriefed about it only after they had left. At face value, this measure was the closest one to behavior in everyday life, since there was no one observing and the situation was as natural as it could be. Although it is not possible to interpret null results, we wonder why these null results were obtained in the measure that we would have considered perhaps the most ecologically valid one of the entire experiment.

Participants completed many questionnaires measuring prosocial behavior and it could be argued that these questionnaires may have pressured the participants to act according to their answers in the following situations (the games, volunteering, and donation). However, unexpectedly, none of the behavioral measures of social cooperation correlated with the reported altruistic behavior in everyday life. This could mean that people overestimate the frequency of altruistic deeds in their life compared to their behavior in laboratory setting, or otherwise provide biased reports of those behaviors. Another explanation could be that our experimental measures taken, although commonly used in laboratory studies on prosocial behavior, are no valid indices of behavior outside the laboratory. We cannot say which of these two explanations holds true, but we do think this important question of ecological validity needs to be focused more in the growing research field on prosocial behavior. At least, no clear demand created by the questionnaires to act in certain way during the behavioral measures was observed. It is also noteworthy that several German versions of the measures have not been validated.

Taken together, we found that subjectively preferred, trait-like, thinking style may affect the decision to share when under observation, to altruistically punish, and to volunteer in a situation that requires moral courage. FI promoted the first two behaviors and need for cognition the third. Our findings are the first to suggest a dissociation of the cognitive-emotional underpinnings of different types of altruism, and may be helpful for designing interventions and campaigns aimed at engaging people into altruistic, prosocial, and cooperative behavior in a world with an ever-growing population whose resources are limited.

## Conflict of Interest Statement

The authors declare that the research was conducted in the absence of any commercial or financial relationships that could be construed as a potential conflict of interest.
